# A Statement of Facts in Regard to the Controversy between the Late Faculty of the Atlanta Medical College and Certain Members of the Georgia Medical Association

**Published:** 1871-05

**Authors:** 


*A Statement of Facts in Regard to the Controversy Between the Late Faculty of the Atlanta Medical College and Certain Members of the Georgia Medical Association.*

---

From various quarters we are informed, that the profession in the State, who have not attended the meetings of the Georgia Medical Association, and, indeed, many of those who have participated in its deliberations during the period since the introduction of the controversy between certain individuals, and the former Faculty of the Atlanta Medical College into that body, are not acquainted with the facts involved in the warfare, which has been made upon the Institution alluded to.

For the greater part of the period referred to, the Faculty of the assailed Institution have quietly folded their arms and allowed their enemies to traduce them, and to make footballs of their reputation at several meetings of the Association, not believing it possible that personal feeling could be carried to such an extreme as was exhibited in the meeting of last year at Macon, when by resolution—a very few participating (we learn not exceeding twelve or fifteen) in the violent action—the names of the said Faculty were stricken from the rolls of the Association.

We have determined to pursue a different policy, and while prepared, under proper circumstances, to admit any error which we may have committed, and to repair any injury which innocent parties may have suffered at our hands, either in reputation or feeling, at the same time to defend ourselves to the full extent of our ability, and, for the future, to maintain our rights at all hazards.

Nothing is better understood in this community, either among the profession or the people, than the fact that the original assault upon the Institution was a *purely personal one*, originating with individuals who, having been connected with it, were *compelled to retire* from *incompetency, unfaithfulness or unprofessional conduct*, or who had been unsuccessful aspirants for position in it.

Unpleasant as it may be for us to designate individuals, the truth of history, and the stern demands of a duty which we have determined to discharge without ceremony, and with a full knowledge of and preparation for any responsibility involved, compels us to state that while there were, in our opinion, ungenerous rivalries manifested toward the Institution, during the earlier years of its existence, upon the part of one or two medical institutions, and particularly upon the part of the Medical College of Savannah previous to the war, the contest in which we have been engaged since that time was commenced by the introduction of a resolution, to discredit the Institution, into the meeting of the Georgia Medical Association in 1868, at Augusta, by Dr. G. G. Crawford, who had been compelled to retire from the position of Demonstrator in the College, in 1866, on account of dissatisfaction upon the part of the class with him, either for unfaithfulness or incompetency.

The pretext for this assault upon the Institution *originated* with Dr. Thomas S. Powell, (who also aided and abetted the said Crawford in the action referred to,) who had been *expelled from the Faculty on account of unprofessional conduct*—and particularly the constant and persistent publication of himself in one or more newspapers in connection with what he called "A Ladies' Home"—as peculiarly skilled in the treatment of the diseases of females, having, indeed, established a newspaper devoted specially to this purpose, thereby rendering himself odious to the profession throughout the State and country, and to a very serious extent damaging the reputation of the Institution. In this connection, the following extract from the records of the Atlanta Medical College are presented:

"August, 31st, 1866.

"*Resolved*, 1*st*. That the course pursued by Prof. Powell in the prosecution of the enterprise he calls the "Ladies' Home" meets with the positive disapprobation of his colleagues in the Faculty of the Atlanta Medical College.

"*Resolved*, 2*d*. That the Faculty, from the information which they have, are satisfied that the medical profession in Georgia generally are opposed to Prof. Powell's course in conducting this enterprise.

"*Resolved*, 3*d*. That the Faculty feel that the interest of the Institution is likely to suffer from the course of Prof. Powell, and that they are disinclined to co-operate with him further as a member of the Faculty; they, therefore, request his resignation."

We also find in the records of the Faculty for November 6th, 1866, in a report to the Trustees, the following statement:

"Since Dr. Powell's connection with the institution, charges of unprofessional conduct have been freely urged against him, and whilst the Faculty felt deeply grieved and mortified that one of their colleagues should be amenable to such charges, still, as the evils complained of were of a local character, and, consequently, local in their influence, they declined taking any official action upon them. But when, in the establishment of the Institution called the "Ladies' Home," the profession abroad became unfavorably impressed toward Dr. Powell, and through him towards the College, the Faculty felt in duty bound, in defence of their cherished Institution and their own reputation, to adopt the course indicated. The occasion does not permit us to give the evidence in detail upon which these accusations are founded, it is enough to know that they are generally entertained, and that the Institution in question, ("The Ladies' Home,") is generally regarded as an imposition upon the public."

Dr. Powell having failed to tender his resignation in accordance with the opportunity afforded him by the forbearance and generosity of the Faculty, (having long borne his notorious professional irregularities,) on November 20th, 1866, adopted the following:

*Resolved*, That in accordance with the action taken by the Faculty and recorded in their minutes of November 6th instant, a copy of which was communicated to the Board of Trustees, we hereby declare the Chair of Obstetrics, Diseases of Women and Children in this Institution, heretofore occupied by Dr. T. S. Powell, now vacated."

The *pretext* alluded to as the ground upon which the charge of irregularity against the College was made by Drs. Powell and Crawford (and instigated by others who had been disappointed aspirants for position in the College, especially Dr. E. J. Roach, who had been a candidate for Demonstrator without success, certainly upon two, probably three, occasions, and who has recently figured as *Secretary pro tem.* in furnishing credentials from the "so-called" Fulton County Medical Society to the State Medical Association to Dr. Powell and Assistant,) was an amendment to the charter obtained in 1858, by which the Faculty were *simply*

allowed the privilege of managing the affairs of the College, instead of trusting them to the care of the Board of Trustees, who had manifested no great interest in the discharge of their duties or in the prosperity of the Institution. An amendment, as we assert, based upon what was understood by the Faculty at the time, and now asserted to be true, giving them only the same power as was being exercised by the Faculty of the Medical Department of the University of Nashville, and which was never designed to violate in any way the regulations of the profession, and which in a period of ten years, during which it had been in existence, had never been used for any irregular purpose. Indeed, the only absolute exercise of any power granted under it, which had not belonged to the Faculty under the old charter, or had not been exercised by them, virtually at least, was the expulsion of Dr. T. S. Powell from the Faculty for unprofessional conduct, thus signally vindicating the wisdom of its provisions.

But as we now hope there were other parties acting under a misapprehension, and influenced by a regard for the honor of the profession, (very sublimated, theoretical and imaginary it is true,) who perpetrated the great injustice of aiding in putting the Institution under the ban of the Association by a most summary measure—not by way of advice, or of affording an opportunity of complying with a friendly admonition—but placing it so far as they had power, beyond the pale of respectable institutions. Not taking action in reference to any offence which had been committed under the charter, but rendering up judgment and putting the seal of professional condemnation and discredit upon the College and its Faculty, as has recently been published to the world; not for the exercise of any irregular power under the charter, but in view of the *possibility* that a number of medical men might exercise improper powers under this plenary clause, which would not be likely to occur in connection with a Board of non-professional Trustees. Or, according to the very language of the most active participants themselves, in this most extraordinary course, in a labored defence which has reached us in the past few days, from the Savannah Medical Society:

"The amended charter [ten years in existence without any offence being charged, or any protest being entered.—Eds.] gave to the Faculty unusual and, as many believed, dangerous powers."

Or, according to another deliverance from the only other Medical Society from which we have heard, that of Macon, "it was just and proper, in view of the great abuses in the matter of graduation to which the amended charter of said College *invites* its Faculty," that the Association should refuse to recognize the College; that is to say, we will not whisper a little friendly advice or admonition in your ear as to the *possibility* of your demoralization, *but we shall at once apply the penalty attaching to the commission of crime that you may be saved from temptation.*

Smarting and intensely irritated under this most unjust and and oppressive course, and aggravated by the introduction of the question into the Legislature of the State, managed and directed by Dr. Powell and others, an offensive memorial was prepared as a *legal* document, by counsel employed in the case, which was signed by a majority of the Faculty without sufficient reflection in the excitement of this bitter contest. While it was true, as already intimated, that very offensive and improper statements were made against individual members of the Georgia Medical Association, who did not reside in this city, and which we now very much regret, there is no doubt that the movement against the College did originate with the individuals referred to as having become hostile to the Institution immediately upon their expulsion from it, and that they deserved the full measure of denunciation contained in the memorial, there is no question whatever.

Notwithstanding, however, the exceedingly aggravating circumstances under which the said memorial was prepared, it was distinctly stated in it that there was no intention to reflect upon the Georgia Medical Association as a body, the design of which was not to convey the idea that there had been no meeting of that body, but that the meeting was not a full one, and not fairly representing the voice and sentiment of the profession in the State, and was composed, to a large extent, of individuals inimical to the College, and of others connected with rival interests, and we now insist that the action taken by that body was *unjustifiable* and not in accordance with the principles of *justice* and fair dealing which would have required the adoption of Dr. Dugas' resolution referring the subject to a committee to report at the next meeting of the Association, thus giving notice to the assailed parties to correct the charter complained of, or appear in its defence.

But, notwithstanding this extraordinary and precipitate action, the Faculty determined, in the interest of harmony, to yield to the voice of the Association thus improperly expressed, never having attached any special importance to the amendment to the charter, except so far as it gave them the power to select their own associates, or, in other words, to elect professors to fill vacancies, and to expel those who were guilty of offences, as had been done in the case of Dr. Powell, the exercise of which was, as is well known, his only objection to the amendment.

In accordance with the above statement, we find that, notwithstanding the Legislature refused to repeal the amendment, and Judge Warner decided that it had been legitimately obtained, and the Faculty were thus sustained by the law making power and the Judiciary, they agreed, according to their record of October 10th, 1868, "that the powers conferred by the charter, and by the amendment thereof, shall be henceforth vested in the Board of Trustees," and united with the Trustees in an application to the Legislature for such change, thus yielding their rights even to this partially expressed will of a very small portion of the profession in the State.

The change in the charter was made, and the original requisition fully met, and the professional question *settled*, as admitted by the adoption of Dr. Charter's resolution at the meeting in Savannah the next year, which declared that "whereas the irregularity of which this Association complained at its last session in the charter and management of the Atlanta Medical College has been removed by the action of the last Legislature, and said College is now conducted upon principles which this Asssociation approve: Be it resolved, That the resolution adopted by the Association, at its last session, discrediting the diplomas of the Atlanta Medical College, shall have no reference to diplomas that may be conferred by that Institution hereafter." It is true that this resolution was adopted with an amendment of Dr. Juriah Harriss, (a member of the Faculty of the Savannah Medical College,) to the following effect: "Provided the present Faculty under the new charter will repudiate the action of the Faculty, existing at the time of the passage of the resolution during the meeting at Augusta," which could not in any sense alter the fact that the Institution was then conducted upon principles which the Association approved, and unmistakably and indelibly fixed upon the future action of those who continued to prosecute this matter, a personal and revengeful character, and this, too, in the face of the fact that at the same meeting and previous to the introduction of this amendment by Dr. Harris, a communication had been received from the Faculty of the Atlanta Medical Col-ege, as follows:

ATLANTA, GA., April 10, 1869.

At a meeting of the Faculty of the Atlanta Medical College of 1868, held in the city of Atlanta, March 16th, 1869, the following resolution was unanimously adopted, viz:

*Resolved*, That this Faculty disavow any purpose to reflect upon the Medical Association of Georgia, either in the memorial presented to the Legislature of 1868, or upon any other occasion, and that our representatives who may attend the meeting of said Association, to be held in the city of Savannah, on the 14th inst., be, and they are hereby, instructed to present this disavowal together with that contained in said memorial.

By order of the Faculty.

JESSE BORING, M. D., *Dean of the Faculty.*

W. S. ARMSTRONG, M. D., *Secretary.*

Thus it will be perceived a second time distinctly and emphatically disavowing any intention to reflect upon the Association, and thus will also be perceived the determined purpose to introduce a new question of an equally distinctly marked personal and private character arising in connection with matters occurring outside of the Association, and so clearly understood to be matters of individual grievance as to justify, in the opinion of those interested in the contest, the adoption of resolutions (unprecedented in a scientific body) making a violent and abusive personal assault upon the veracity and honesty of purpose of the "men" who had published the memorial. Not satisfied with the second disavowal, a resolution is adopted requiring not only a distinct and unequivocal withdrawal of the objectionable language used in a *non-professional* paper (published *outside* of the Association, with the distinct declaration accompanying that it is not to be understood as applying to the Association, *but to individuals*,) but that such withdrawal must be published in the *newspapers* of the State, thus distinctly again exhibiting a temper and spirit entirely foreign to a scientific body, and affording unmistakable evidence of the *personal* animus pervading the movement.

Still determined, if possible, to repair any wrong which may have been committed during the excitement alluded to, the Faculty determined to go still farther, and forwarded through the Board of Trustees to the meeting of the Association, in Macon, last year, (the succeeding session,) the following not only full and explicit disavowal of any intention to reflect upon the Association, but absolute *retraction* and withdrawal of anything contained therein that might be liable to an offensive construction :

ATLANTA, GA., August 25, 1869.

*Gentlemen:* I have the honor to acknowledge your note of the 23d instant, as a committee of the Trustees of the Atlanta Medical College. In reply, I am instructed to convey to you the assurance of a sincere desire on their part to reach a satisfactory and honorable termination of the misunderstanding between them and the Medical Association of Georgia, and their willingness to make such explanation of the memorial complained of, as will satisfy honorable gentlemen.

The language of the memorial objected to by the Association was not, we are assured, intended to be applied to it, but to other parties, whose attacks upon the late Faculty it was thought necessary to repel, and it is only, we believe, by an erroneous construction that such an idea could be entertained; but so careful of misconstruction were the authors of the memorial, that they accompanied its publication in the newspapers and in the pamphlet form with a note disclaiming all intention to reflect on the Medical Association. This disclaimer unasked, they repeated in a a more earnest and solemn form in a resolution laid before the Association at its last meeting, and we take the occasion to state that in so far as any of the authors of that memorial are members of the existing Faculty, none of the statements made or opinions expressed therein were intended to apply to the Georgia Medical Association. To the extent that the said memorial may be liable to any other construction, it is cheerfully retracted.

This explanation is given simply because it is true, and being true, is due the Association from the members of the late Faculty. So far as the language of the memorial refers to individuals, the late Faculty having ceased to exist as an organization, can only respond as private persons to the parties aggrieved.

In relation to the action of the Georgia Medical Association at Savannah, the present Faculty of the Atlanta Medical College rejoices to learn from their minutes that said Association has declared the College to be now conducted upon principles approved by the Association; and in relation to the proviso to their resolutions, the Faculty hereby assert their hearty sympathy with the Association and the full approval of the repeal of the amendment of 1858 to their charter, and will in the future conform in all respects to the spirit of the Association and the profession.

I am, gentlemen,

You obedient servant,

JESSE BORING,

*Dean of the Faculty, &c.*

By order of the Faculty.

Messrs. C. L. REDWINE, W. M. LOWRY, W. W. CLAYTON, Committee, &c., Atlanta, Ga.

This paper was so far from being satisfactory, (with the then determined purpose of the ruling spirits to degrade the individuals involved,) that it was not even permitted a place upon the records of the Association, and is even not found in what professes to be a history of the troubles, lately issued by the Medical Society of Savannah, a number of the members of which have been—outside of the small faction in this city—our most persistent and unrelenting persecutors.

It must be recollected that this work has been done mainly by those who originated the movement in this city, and those who, however pure their motives in the commencement, undoubtedly labored under a misapprehension or committed a most grievous error in the violation of vested rights, not in any way in conflict with the regulations of the medical profession, and afterwards becoming involved in a personal difficulty with those they had wantonly assailed, manifested a purpose of determined persecution which could only have originated in a spirit of retaliation and revenge, which finally culminated in an exercise of accidental power in the way of punishment for assumed grievances which could not, by any standard of right, have been the subject of such action as that exhibited in the expulsion of the former Faculty of the College at the Macon meeting.

In addition to all this, and immediately after the action in Macon, the American Medical Association threw the whole question out of that body—into which it had been introduced by those who were prosecuting it within the Georgia Medical Association—thus continuing its relations with the College, as they had previously existed, upon the same footing with the other Medical Institutions of the country, and this, too, in face of the partisan representations of "men more bent upon selfish purposes than with a view to elevate the standard of their profession."

Notwithstanding, however, this action of the American Medical Association declaring that there was no existing ground for complaint against the College, (the feature complained of having been corrected by the Legislature upon application of the Faculty,) and their own action in Savannah, admitting that the College was then conducted upon principles which they approved; the Atlanta faction continued to agitate the question in this city, and throughout the State, by every possible means, aided and abetted by (as we have reason to believe) the individuals in Savannah who had already been so active and malignant in their assaults upon the former Faculty of the College. This agitation assumed the shape of a refusal to recognize as medical men, entitled to the ordinary courtesies of the profession, all those who even extended the privileges of consultation to those who had been so unjustly dealt with at the Macon meeting by (as was stated upon the floor of the late meeting at Americus and not denied) some twelve or fifteen *voting* members of the Georgia Medical Association, acting in the name and for the time accidentally exercising the powers of that body. Not satisfied with the outrage perpetrated at the Macon meeting, the faction in this city already designated, in connection with the Savannah representatives, endeavored to exclude all the permanent members of the Association, and, of course, all other medical gentlemen from this city who had continued to recognize the outraged members of the Association as *physicians.* This was done by a paper, *false* in statements and malignant in spirit, purporting to emanate from what was called the "Fulton County Medical Society," a concern not having a sufficient number of members, according to the Constitution of the State Association, to entitle it to representation or recognition as a regularly organized medical society, holding its meetings, it is believed (if regularly held) solely for the accomplishment of the unworthy purposes to which such frequent reference has been made.

In their attempt to perpetrate this additional outrage they were most actively assisted by the accidental presiding officer of the body, Dr. J. G. Thomas, of Savannah, acting in the absence of the President, by virtue of his position as Vice-President, and in the interest of the parties from Savannah who had been the most prominent co-operators with the Powell faction in the various steps of the persistent persecution to which we have called attention.

But, fortunately for the ends of justice and right, the sceptre had departed from the hands of the individuals who had so unworthily wielded it, and instead of having the Association composed of those who had introduced this personal matter into the body, and those who had co-operated from other unjustifiable or improper motives, and those who so little understood or were too indifferent to the question to take any part in the defence of *absent members at the three previous meetings, more than two-thirds* of the body were found determined that an investigation of these meetings should be had, and, after two days of the most latitudinous discussion, and the greatest possible indulgence in hearing everything that could be said in defence of the violent action, decided substantially that the whole controversy had been a *personal* one from its introduction into the Association and during every step of its progress, and, being of this character, should never have been introduced into the Association, and all action in regard thereto was rescinded, and declared so entirely antagonistic to the true objects of the organization, and the honor and dignity of the profession, as not to deserve a place in the archives of the body.

The meeting of the Association at Americus was a large one, and composed of gentlemen from every section of the State, and certainly equal in intelligence and in character to the average meetings of the body, and in point of representation from all the different sections of the State more nearly just and equal than any previous body since the termination of the war between the States, or the commencement of the war in the Association.

Notwithstanding the general conviction that a great outrage had been committed upon members of the Association at the previous meetings referred to, and that there had been an entire perversion of the high ends and objects for which the body had been organized, there was manifested on the part of the majority the most generous spirit of forbearance in declining to exercise their power in any spirit of retaliation, and only so far as was regarded as indispensable in protecting the body from the possibility of any future introduction of this or any similar personal matter into its deliberations.

We are sure that no unprejudiced individual can read this statement of facts without endorsing the correctness of the decision of the Georgia Medical Association at its last meeting, as follows:

"Whereas, the controversies between the Faculty of the Atlanta Medical College, the Atlanta Academy of Medicine, the Fulton County Medical Society, or members of the same, who are members of this body, or between said bodies or individuals, and other members of this Association, are of a personal character, and ought never to have been introduced into the Association; therefore,

"*Resolved*, That all action of the Association upon these controversies be rescinded, and be no longer regarded as a part of the archives of this body."

And yet the parties to this contest who had originally introduced it into the Association, or who had operated with them and who had controlled it so long as the assailed individuals quietly submitted to their wrongs and made no effort to defend their rights, (trusting to the hope of a returning sense of justice and right, and not realizing the possibility of such extremes,) as soon as a tardy justice is obtained, commence the attempt to disorganize the body and to throw discredit upon those who composed the last meeting, even to the extent upon the part of the Savannah gentlemen of declaring in substance "that the honor and dignity of the profession can only be upheld by replacing the Association in our hands, and though there is no proper appreciation of these things, outside of our "ancient and beautiful city," for the time being to regain our lost prerogative, and to save the profession from degradation, we will consent to meet a few of the more decent from Macon and Augusta, and even the few for whom we have the most profound contempt, who have aided us from Atlanta, for the purpose of condemning the action of the late meeting of the Georgia Medical Association, and furnishing an authoritative command to the next body of "men" who are to assemble next year in Columbus, to surrender without a word to our demands for the control of the medical affairs of the State."

These very parties who have proclaimed so loudly and authoritatively the "dignity and honor," the power and right of absolute and boundless control belonging to the Georgia Medical Association while in *their hands*, now seriously and gravely (and naively) propose a Convention of the medical men of the State in Macon, on the 5th July, for the purpose of condemning the late action of the Georgia Medical Association, and repairing its honor and dignity, and that of the profession which had suffered violence at the hands of the same medical men—knowing, as they do full well that no one will attend such meeting, as already intimated, under such call, except those who are already committed to a partisan action in behalf of these self-constituted guardians of the "dignity and honor" of the profession, and who will simply meet to register the edict of Savannah professional jealousy and arrogance, overshadowing as it does the Powell faction of this city.

Nothing would be more agreeable to us in this connection, notwithstanding the great wrongs we have suffered at the hands of these parties, than to meet the entire profession of the State, and, after a full and thorough exhibition of the facts, in a spirit of compromise and concession, submit to their verdict, whatever it might require of us in the way of apology for or retraction of anything we may have said by which any honorable man may been injured or wounded, and whenever there is the slightest manifestation of a desire to settle amicably and honorably any or all of the existing differences, that disposition will be more than duplicated by those who (whatever wrongs they have committed during the progress of this controversy) have certainly not been the *aggressors*.

We have failed to state in its proper connection an important fact bearing upon this controversy and the late proceedings of the Georgia Medical Association at Americus, to this effect, that a number of the permanent members of that body who countenanced or justified the introduction of the question at issue into the meeting at Augusta, have long since become informed of the true nature of the controversy, and are among the warmest friends of the late Faculty. Besides we have good reason to believe that a number of those who acted with the minority in their efforts to prevent the settlement proposed and adopted at the late meeting, are determined to accept the action as final, and will not consent to the introduction of this question again into the deliberations of the Georgia Medical Association, which, according to our Savannah *friends*, has occupied the time of the Association for the past four years, to the exclusion of legitimate business.

In conclusion, we have the great gratification of asserting that with a very few exceptions, we are sustained to the fullest possible extent by every respectable medical man of this city, and we have reason to believe by the large mass of the profession in the State, outside of two or three towns where there are rival interests to sustain, or where the professional men have partaken of the envious and jealous feelings pervading these localities toward the rapidly growing interests of this progressive city. And, as a last word, we would say that this exhibition of truth has afforded us no pleasure, and nothing but the stern necessities of *self-defense*, under the most aggravating circumstances, could have induced us to enter upon so distasteful a task; at the same time we desire it to be understood that having entered upon this work, after a long delay of waiting for justice from other sources, we shall not fail in the future to demand and maintain our rights and position, let the consequences be what they may.

